# Chirality-Driven Electronic, Mechanical, and Hydrogen Adsorption Properties of Dodecanophene Nanotubes

**DOI:** 10.1021/acsomega.5c07529

**Published:** 2026-01-22

**Authors:** Juan Rafael Gomez Quispe, Fernando Guido Ordinola Sanchez, R. M. Guzmán-Arellano, Chachi Rojas-Ayala, Pedro Alves da Silva Autreto

**Affiliations:** † Center of Natural and Human Sciences, 74362 Federal University of ABC, Santo Andre, Sao Paulo 09210-580, Brazil; ‡ Facultad de Ciencias Físicas, 33209 Universidad Nacional Mayor de San Marcos, Lima 15081, Peru

## Abstract

We report a detailed theoretical investigation into the electronic, mechanical, and hydrogen adsorption behaviors of zigzag dodecanophene nanotubes (Dode-NTs) with chiralities (n,0) and (0,n). Using density functional theory (DFT) and classical reactive molecular dynamics (MD) simulations, we demonstrate that chirality and curvature strongly modulate the physical behavior of these nanotubes. The Dode-NTs (n,0) maintain a robust metallic character even under uniaxial strain, whereas Dode-NTs (0,n) with odd chiral indices exhibit a tunable semiconducting behavior, with frontier orbitals spatially separated along transverse and longitudinal directions. Mechanically, Dode-NTs (n,0) exhibit higher stiffness and tensile strength, confirmed by both DFT and MD, while Dode-NTs (0,n) show a more ductile response with distributed strain accommodation. These features highlight a pronounced mechanical anisotropy. The hydrogen adsorption studies reveal that the Dode-NTs (n,0), particularly at a specific adsorption site and at larger diameters, exhibit adsorption free energy values near the catalytic optimum for the hydrogen evolution reaction (HER). In contrast, Dode-NTs (0,n) present lower reactivity and weaker site selectivity. MD results confirm a more efficient surface functionalization for the Dode-NTs (n,0) configuration under elevated temperatures. These findings highlight that Dode-NTs, especially those with (n,0) chirality, are highly tunable nanostructures with potential applications in catalysis, hydrogen storage, nanoelectronics, and nanomechanical systems.

## Graphical abstract

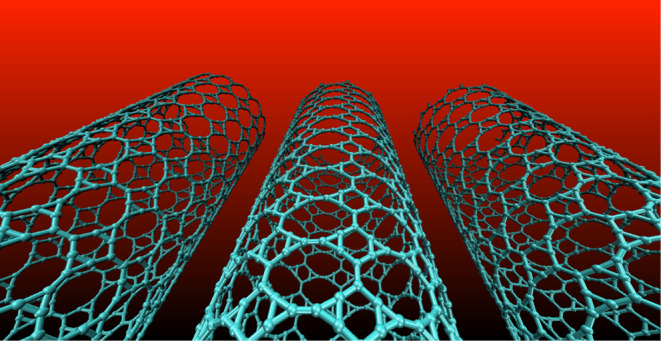

## Introduction

The rapid advancement of two-dimensional (2D) carbon-based materials has significantly stimulated research into their one-dimensional (1D) derivatives, such as nanoribbons and nanotubes, due to their promising applications in electronics, mechanics, energy storage, and optoelectronics. Unlike graphene, which consists exclusively of hexagonal (*C*_6_) rings formed by carbon atoms in a planar *sp* ^2^ hybridization, emerging 2D carbon allotropes incorporate diverse carbon ring sizes, leading to complex and porous topologies. This structural variability allows the design of nanotubes derived from these novel allotropes, opening a new avenue toward advanced materials with multifunctional characteristics suitable for various technological fields.

Recently, nanotubes proposed from these new 2D carbon allotropes such as X-graphene,^1^ Y-graphene,^1^ S-graphene,^2^ twin graphene,^3^ HOP-graphene,^4^ C-57 graphene,^5^ TPDH-gr,^6^ and PAI-G,^7^ among others, have demonstrated remarkable structural and dynamic stability. Depending on their carbon ring topology, chirality, and curvature, these nanotubes exhibit metallic, semimetallic, or semiconducting behaviors. For instance, nanotubes based on R_12_-graphene (R_12_-GNT), composed of *C*_4_, *C*_5_, *C*_6_, and *C*_12_ rings, display anisotropic mechanical properties, particularly enhanced fracture resistance and efficient stress dissipation in the zigzag configuration.^8^ Similarly, phagraphene-derived nanotubes (PhaNTs), comprising *C*_5_, *C*_6_, and *C*_7_ rings, achieve Young’s modulus up to 916 GPa and ultimate tensile strengths of approximately 250 GPa, underscoring their potential for structural applications under extreme mechanical conditions.^9^

Among these promising 2D allotropes, dodecanophene has recently emerged as a stable porous network consisting of *C*_3_, *C*_6_, and *C*_12_ carbon rings. This material exhibits metallic characteristics with robust Dirac cones and significant optical activity spanning from infrared to ultraviolet wavelengths, without alterations in electronic behavior under applied strain.^10^ Molecular dynamics (MD) simulations have revealed highly anisotropic mechanical responses of dodecanophene nanosheets, influenced significantly by their dimensions, temperature, defects, and stacking configurations, with Young’s modulus ranging from 409 GPa (armchair direction) to 592 GPa (zigzag direction).^11^

Although dodecanophene is currently a theoretical carbon allotrope whose predicted properties arise from computational modeling and await experimental validation, recent advances in on-surface, bottom-up synthesis have yielded 2D carbon allotropes with ring-rich architectures and extended *C* – *C* chains such as the biphenylene network,^12^ MAC,^13^ and α-graphyne^14^ that closely mirror the structural motifs proposed for dodecanophene. These precedents make dodecanophene-like topologies a realistic experimental target. Moreover, combining such 2D syntheses with template-assisted roll-up or capillary scrolling offers plausible routes to tubular analogues while preserving the complex distribution of carbon rings.^15–17^

Building on these precedents, it is important to acknowledge key experimental challenges. First, precursors containing *C*_3_ rings are intrinsically strained and therefore prone to ring-opening or rearrangement during deposition and on-surface coupling; their controlled handling on metal surfaces requires careful selection of the substrate, leaving group, temperature, and transient organometallic states, as demonstrated even for highly reactive carbon allotropes generated on surfaces.^18–20^ Second, converting 2D sheets into tubes via roll-up/capillary scrolling demands simultaneous control of curvature and crystallographic registry, which governs chirality, defect density, and yield; strategies such as pre-imposed mechanical anisotropy (wrinkling), microdroplet-guided delamination/intercalation, and reproducible graphene nanoscroll protocols exemplify viable control routes.^21–23^ This brief note is not meant to exhaust the topic, but to provide a balanced context for the prospective experimental realization of Dode-NTs.

Nanotubes derived from other novel 2D carbon allotropes, such as PAI-G and TPDH-gr, preserve desirable electronic features (metallicity and strain-tunable band gaps) while displaying outstanding mechanical properties, with Young’s modulus above 700 GPa, tensile strengths over 80 GPa, and high deformation capacity.^6,7^ C-57 nanotubes further demonstrate how curvature enhances electrical conductivity and optical performance.^5^ In terms of energy storage, alkali-decorated allotropes like TPHE- and TPH-graphene show excellent hydrogen and metal-ion storage capabilities.^24,25^ Notably, Na-doped TPHE-graphene achieves hydrogen storage capacities above 9.5 wt % with thermal stability and metallic conductivity,^24^ while KT-graphene exhibits strong K-ion adsorption and ultralow diffusion barriers, making it promising for potassium-ion battery anodes.^26^

Nanotubes derived from X- and Y-graphene with ZZ stacking achieve elastic modulus up to 1192 GPa and tensile strengths of 201 GPa, underscoring the role of structural engineering in enhancing mechanical performance.^1^ Twin graphene nanotubes maintain Young’s modulus above 300 GPa for diameters up to 10 nm, showing stability under extreme conditions.^3^ Moreover, HOP-graphene nanotubes exhibit polymer adsorption energies up to 268 times higher than conventional CNTs, making them promising for functional interfaces and advanced composites.^4^

Unlike conventional CNTs and the nanotubes formed from other 2D sheets discussed above, dodecanophene nanotubes (Dode-NTs) intrinsically host three-membered (*C*_3_) carbon rings along their porous rims. These *C*_3_ motifs impose pronounced angular strain and local pyramidalization, promoting σ–π rehybridization, and giving rise to intrinsically active catalytic centers whose presence does not depend on chirality. In contrast to physisorption, which involves noncovalent interactions dominated by van der Waals forces and plays a crucial role in hydrogen storage, hydrogen evolution catalysis relies on covalent interactions occurring at reactive sites.

Therefore, in the context of the hydrogen evolution reaction (HER), this architecture provides sites with enhanced H affinity, enabling a structure-driven activation pathway that does not rely on metal dopants or induced defects. To our knowledge, the specific role of *C*_3_ rings in porous nanotubes has not been systematically assessed.

In this work, we present the first comprehensive theoretical study of zigzag Dode-NTs, (n,0) and (0,n), using density functional theory (DFT) and classical reactive molecular dynamics (MD). We isolate how chirality and curvature (tube diameter), together with intrinsic three-membered carbon rings (*C*_3_) along the porous rims, which governs the mechanical response under uniaxial tension, the electronic structure that includes band gaps and band edge localization, and the hydrogen adsorption relevant to HER within the computational hydrogen electrode framework. This integrated view supports Dode-NTs as metal-free, multifunctional candidates for structural, electronic, and electrocatalytic applications.

While the present study focuses on zigzag Dode-NTs ((n,0) and (0,n)) to establish baseline chirality and curvature trends, extending the analysis to truly chiral (n,m) tubes is a natural next step toward a comprehensive geometry-property map. Practically, zigzag and armchair tubes define high-symmetry limits that bracket electronic (armchair metallic; zigzag predominantly semiconducting with curvature-induced gaps) and mechanical extremes; starting with zigzag thus provides a symmetry-simple baseline for chirality-dependent trends and sidesteps axial/rotational commensurability mismatches that preclude compact common supercells and substantially increase computational cost.

## Computational Methods

Dodecanophene nanotubes (Dode-NTs) were constructed using Crystallographic Information Files (CIF files) of the previously optimized planar structure obtained through density functional theory calculations. Nanotube models with zigzag chiralities (n,0) and (0,n) were generated using the Cif2Tube software,^27^ which applies coordinate transformation algorithms to roll the 2D sheet into tubular geometries.^6,27^ Figure 1a,b displays the Dode-NTs with chiralities (15,0) and (0,11), respectively. A distinct pore distribution can be observed in each case, arising from the different chiral configurations.

**Figure 1:**
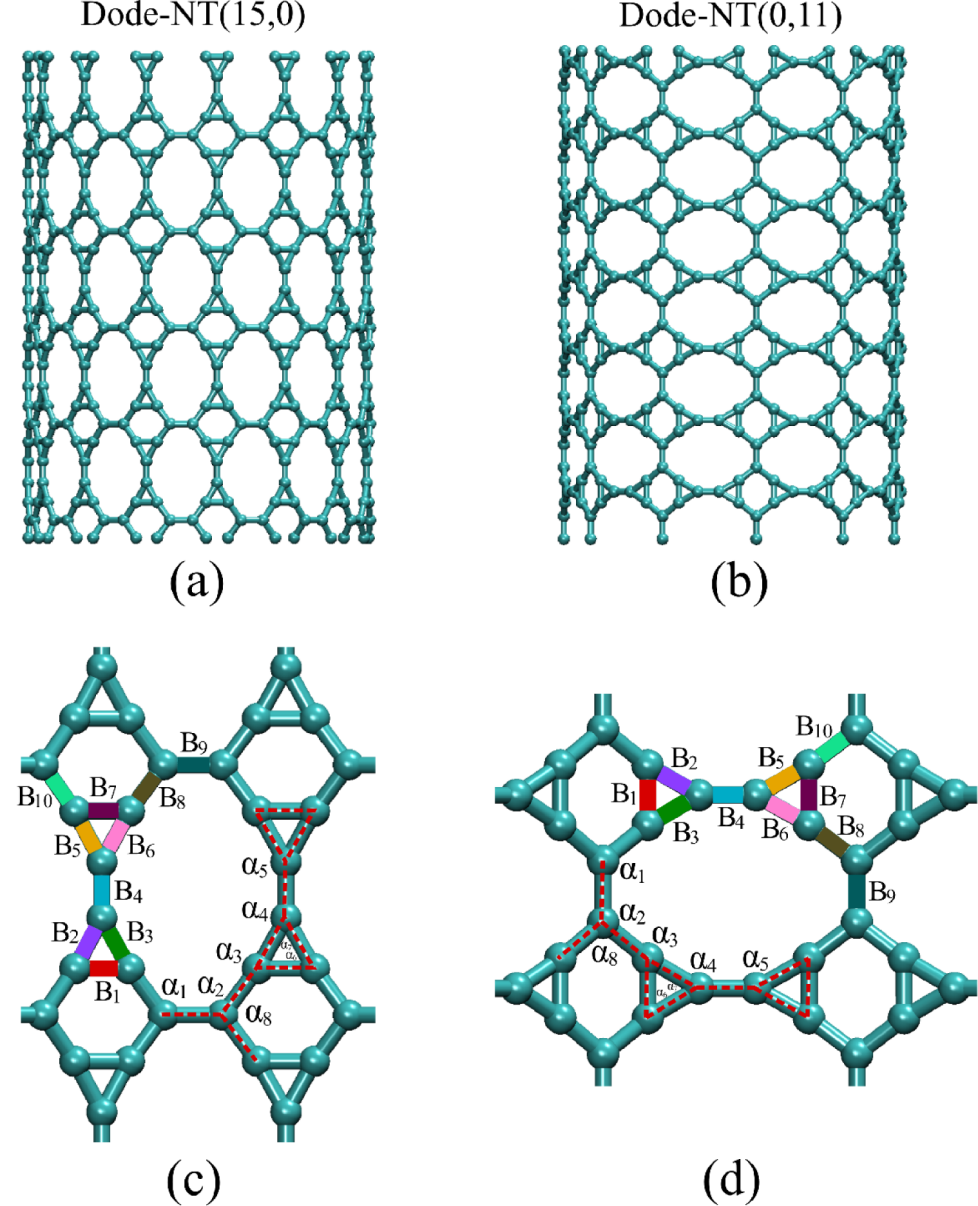
(a) Zigzag Dode-NT (15,0) configuration. (b) Inverse zigzag Dode-NT (0,11) configuration. (c, d) Typical bond lengths and bond angles in the structure of Dode-NTs.

As previously mentioned, dodecanophene is composed of *C*_3_, *C*_6_, and *C*_12_ carbon rings that together form a uniformly porous topology. This framework exhibits characteristic bond lengths and bond angles, as illustrated in Figure 1c,d, which shows the typical structural parameters for the conventional unit cell of dodecanophene, oriented along the horizontal and vertical directions, respectively. These structural parameters are essential for understanding the mechanical behavior and deformation mechanisms of Dode-NTs.

### Density Functional Theory

Density functional theory (DFT) was employed to investigate the stability and electronic properties of Dode-NTs. Calculations were performed using the SIESTA code, which utilizes a set of localized numerical atomic orbitals to represent the electronic wave functions.^28,29^ In this work, a double-ζ polarized (DZP) basis set was employed, extracted from the SIMUNE pseudopotential database,^30^ which ensures a balanced description of valence states. This approach allows efficient description of large systems. Additionally, SIESTA implements norm-conserving Troullier–Martins pseudopotentials in the Kleinman–Bylander form, which simplifies the treatment of the core and inner-shell electrons, reducing computational cost without compromising accuracy.^31^ The exchange-correlation interactions were described using the generalized gradient approximation (GGA) in the Perdew–Burke–Ernzerhof (PBE) formulation.^32^

For all calculations, a mesh cutoff of 350 Ry was used to define the real-space integration grid, and a force convergence threshold of 0.02 eV/Å was applied during structural relaxation. During each relaxation step, the self-consistent field convergence criterion for the total electronic energy was set to 10^–4^ eV. A Monkhorst–Pack^33^ *k*-point mesh of 1 × 1 × 12 was employed for the calculation of the electronic band structure. Band structures correspond to 0 K static-lattice DFT calculations; finite-temperature gap renormalization (electron–phonon coupling and thermal expansion) is not included, and a quantitative assessment at 300 K lies beyond the scope of this work.

Dispersion (vdW) corrections were not included because, in *sp* ^2^ carbon structures, hydrogen adsorption and the axial mechanical response are dominated by covalent *C* –*H* and *C* –*C* bonding, respectively. For isolated single-wall nanotubes separated by a large vacuum region, vdW interactions mainly provide a small, nearly uniform energy shift and only minor corrections to equilibrium bond lengths.^34,35^ Consequently, all DFT calculations, including geometry optimizations of pristine and hydrogenated nanotubes and stress–strain simulations, were carried out without explicit vdW corrections; under these conditions, relative trends across adsorption sites, chiralities, and tube diameters, evaluated consistently at the same level of theory, are not expected to be affected within our reported energy resolution.

When a dodecanophene sheet is rolled into a tubular structure, intrinsic strain arises due to the imposed curvature. This strain can be quantified by the curvature energy (*E*_curv_), defined as^6,36,37^

1 $$E_{\mathrm{curv}}=E_{\mathrm{tube}}-E_{\mathrm{sheet}}$$

where *E*_tube_ and *E*_sheet_ represent the total energies per atom of the Dode-NT and its corresponding planar sheet, respectively. The curvature energy serves as an essential metric to evaluate the thermodynamic feasibility of nanotube formation, providing insight into the energetic penalty associated with bending the flat structure into a tubular geometry.^6^

The effect of longitudinal strain on the electronic properties of Dode-NTs was investigated using a quasi-static approach.^6,38^ The strain energy was evaluated by applying incremental vertical elongations to the nanotubes, with the applied strain defined as ϵ_*z*_ = *L*/*L*_0_, where *L*_0_ and *L* denote the equilibrium and strained lengths of the nanotube, respectively. For each strain value, full structural relaxation was performed until the maximum force component on any atom was less than 0.02 eV/Å.

Additionally, to determine the elastic properties using DFT calculations, the Dode-NTs were modeled as rolled membranes with a uniform wall thickness of *h* = 3.35 Å and a surface area given by π*d*_*t*_ *L*_0_, where *d*_*t*_ denotes the nanotube diameter and *L*_0_ the equilibrium length. The choice of *h* = 3.35 Å follows the standard convention of adopting the interlayer spacing in graphite, a practice widely used for carbon nanotubes and related nanostructures. This assumption provides a consistent definition of the effective wall volume, facilitating direct comparison with previous studies, while recognizing that the value is a conventional parameter rather than an intrinsic property of Dode-NTs.

Accordingly, the longitudinal stress component σ_*z*_ is related to the strain *ε*_*z*_ by the expression:^6,38^

2 $$\sigma_{z}=\frac{1}{\Omega }\left(\frac{\partial U}{\partial \varepsilon_{z}}\right)$$

where Ω = *L*_0_π*d*_*t*_ *h* represents the effective volume of the nanotube membrane. The Young’s modulus *Y* is obtained from the slope *d*σ_*z*_/*dε*_*z*_ of the stress–strain curves within the linear elastic regime (<3%).

To investigate the interaction between a hydrogen atom and Dode-NTs with chiralities (n,0) and (0,n), the binding energy (*E*_b_) was calculated. This quantity is defined as the energy required to break the C*–*H* bond formed after the adsorption process. Negative binding energies exceeding 1 eV in magnitude indicate a strong covalent character of the bond, whereas positive bindings energies suggest that C*–*H* bond formation is thermodynamically unfavorable.

The binding energy is computed as follows:^39–41^

3 $$E_{b}=E_{\mathrm{NT}+H}-E_{\mathrm{NT}}-E_{H}$$

where *E*_NT+H_ is the total energy of the Dode-NT with an adsorbed hydrogen atom, *E*_NT_ is the energy of the pristine dodecanophene nanotube, and *E*_H_ corresponds to the energy of an isolated hydrogen atom.

The adsorption free energy (Δ*G*_ads_) represents the change in Gibbs free energy when a hydrogen atom becomes adsorbed onto a surface. This parameter is essential for evaluating the catalytic performance toward HER, as it reflects the thermodynamic balance between hydrogen binding and subsequent desorption as molecular hydrogen (*H*_2_).^42–44^ An optimal value of Δ*G*_ads_ close to 0 eV indicates a binding strength that is neither too strong nor too weak, an ideal condition for efficient HER catalysis. Highly negative values imply excessively strong adsorption, which hampers *H*_2_ release, while positive values indicate weak hydrogen binding and thus low catalytic activity. The adsorption free energy was computed using the following expression:^42–44^

4 $$\Delta G_{\mathrm{ads}}=E_{\mathrm{NT}+H}-E_{\mathrm{NT}}-\frac{1}{2}E_{H_{2}}+\mathrm{ZPE}-T\Delta S$$

where *E*_NT+H_ and *E*_NT_ are defined as previously described, $E_{H_{2}}$ is the total energy of a gas-phase hydrogen molecule, and ZPE and *T*Δ*S* denote the zero-point energy and entropy corrections at standard conditions (298 K). These were approximated to a combined value of 0.24 eV,^42^ a practice widely used in DFT studies of HER because adsorption largely suppresses translational and rotational degrees of freedom, leaving the entropy dominated by vibrational modes, and H vibrational frequencies vary only slightly between metal surfaces and typical sites at low coverage.^45–47^ We note that treating the combined ΔZPE–*T*Δ*S* term as a uniform 0.24 eV is an approximation adopted to ensure internal consistency across adsorption sites and chiralities; because these contributions depend on the local vibrational spectrum, future work will determine site-specific corrections from explicit harmonic frequency calculations.

Activation barriers for the Tafel (H* + H* → *H*_2_) and Heyrovsky (H* + *H* ^+^ + *e* ^–^ → *H*_2_ + *) steps were not computed; a rigorous kinetic treatment would require NEB/CI-NEB calculations under constant potential with explicit water layers and coverage-dependent sampling, followed by microkinetic modeling, which lies beyond the scope of this work.

To validate the chosen cell size, we compared adsorption energies for the Dode-NT (0,5) using (1 × 1 × 1) and (1 × 1 × 2) supercells, finding a variation of approximately ∼0.02 eV. This small difference was considered negligible, and thus all calculations were performed with the (1 × 1 × 1) setup for computational efficiency. All adsorption calculations were performed in the low-coverage limit (one *H* per cell). An axial supercell check (1 × 1 × 1 vs 1 × 1 × 2) showed finite-size errors ≤0.02 eV; nevertheless, higher coverages (*n* > 1 *H*/cell) can shift Δ*G*_*ads*_ and the electronic structure via lateral *H* – *H* interactions and coverage-dependent vibrational/entropic effects. A systematic assessment of coverage effects is left for future work.

Charge density differences were obtained as Δρ­(*r*) = ρ­(NT + H) – ρ­(NT) – ρ­(H), evaluated for the relaxed NT+H complex, the frozen bare NT (H removed), and an isolated H atom in the identical cell and grid; symmetric iso-values (0.002 eV/Å^3^) were used in all renderings.

### Molecular Dynamics Simulation

To explore the mechanical behavior of Dode-NTs, we conducted classical reactive molecular dynamics (MD) simulations at room temperature (*T* = 300 K) using the ReaxFF reactive force field as implemented in the LAMMPS package.^48^ ReaxFF is an advanced interatomic potential specifically developed to accurately capture the dynamic formation and rupture of chemical bonds.^49,50^ This reactive nature is particularly important for modeling mechanical responses beyond the elastic limit, including plastic deformation and fracture processes. While other reactive potentials, such as AIREBO-M,^51^ are commonly used to simulate the mechanical and thermal behavior of carbon-based nanostructures, they often require careful parameter tuning to appropriately describe bond-breaking phenomena. In contrast, ReaxFF provides a more transferable and reliable framework for simulating chemical reactivity without the need for system-specific adjustments.

We used the Water/Pt/Ni/Nafion ReaxFF parametrization for all MD simulations.^50^ In our purely *C*–*H* systems, only the *C*–*C* and *C*–*H* terms of this potential are active; these are inherited from the standard ReaxFF hydrocarbon parametrization and have been widely applied to bond breaking and formation in carbon-based materials. Because the study also examines hydrogen chemisorption, using a reactive bond-order potential ensures a consistent treatment of *C*–*C* rehybridization and *C*–*H* formation throughout. A benchmark stress–strain test for Dode-NT­(10,0) (Figure S1) shows that AIREBO-m and ReaxFF match the DFT-derived elastic slope (ϵ ≤ 0.02); at larger strains, AIREBO-m continues hardening and predicts later fracture, whereas ReaxFF exhibits staircase softening and earlier failure. Accordingly, we base failure and adsorption coupled trends on ReaxFF and use the linear regime to validate the elastic modulus against DFT.

To simulate the mechanical response under uniaxial tension, each Dode-NT configuration, zigzag (n,0) and inverse zigzag (0,n), was replicated along the longitudinal (z) direction to achieve a total length of approximately 100 Å. This replication ensured a sufficiently large simulation cell to reduce finite-size effects and allowed for a fair comparison among different nanotube geometries. Given the number and size of the systems involved, performing such mechanical studies using ab initio DFT methods would be computationally prohibitive. Therefore, we employed reactive molecular dynamics simulations as a practical and accurate alternative.

In the present work we restrict our mechanical analysis to uniaxial tensile loading of pristine Dode-NTs at *T* = 300 K, in order to establish baseline chirality and curvature-dependent trends. A systematic investigation of other loading modes (torsion, bending, compression/buckling), explicit temperature dependence, and the role of defects along the tube would require an extensive additional simulation effort and is therefore left for future work.

To eliminate any residual stress before the stretching stage, the nanotubes were first equilibrated through a thermalization procedure using an isothermal–isobaric (NPT) ensemble.^52^ The pressure was set to zero, resulting in a minimal volume variation between 1.0 and 3.0%. All simulations were performed at 300 K, controlled via a Nosé-Hoover thermostat,^53^ and a vacuum spacing of 20 Å was applied around the structures to avoid spurious interactions.

Tensile deformation was introduced by progressively increasing the length of the simulation box along the periodic *z*-direction. The system was evolved in steps of 0.25 fs, with a total simulation time of 1.4 ns, corresponding to a constant strain rate of approximately 0.25 ns^–1^.^54^ The elastic behavior was quantified by calculating the Young’s modulus (Y), estimated as the slope of the stress–strain curve:^6,38^

5 $$Y=d\sigma_{ii}/d\epsilon_{ii}$$

where σ_*ii*_ denotes the longitudinal component of the virial stress tensor and ϵ_*ii*_ represents the longitudinal strain.

The atomic-level stress tensor σ_*ij*_ was computed using the standard virial expression:

6 $$\sigma_{ij}=\frac{\overset{N}{\underset{k}{\sum }}m_{k}\nu_{k_{i}}\nu_{kj}}{V}+\frac{\overset{N}{\underset{k}{\sum }}m_{k}r_{k_{i}}\cdot f_{kj}}{V}$$

Here, *V* = *A* · *h* = *L*_0_ · π · *d*_*t*_ · *h* is the effective volume of the Dode-NTs (considered as a tubular geometry), where *L*_0_ and *d*_*t*_ are the tube length and diameter, respectively, and *h* = 3.35 Å is the assumed wall thickness of the nanotube.^6,55^

To characterize the local stress distribution, the von Mises stress (σ_*VM*_) was calculated for each atom according to the expression:^6,55^

7 $$\sigma_{\mathrm{VM}}^{i}={\{\frac{1}{2}[{\left(\sigma_{xx}^{i}-\sigma_{yy}^{i}\right)}^{2}+{\left(\sigma_{yy}^{i}-\sigma_{zz}^{i}\right)}^{2}+{\left(\sigma_{zz}^{i}-\sigma_{xx}^{i}\right)}^{2}+6\left({\left(\sigma_{xy}^{i}\right)}^{2}+{\left(\sigma_{yz}^{i}\right)}^{2}+{\left(\sigma_{zx}^{i}\right)}^{2}\right)]\}}^{1/2}$$

This stress metric was used to qualitatively evaluate the spatial distribution and temporal evolution of stress accumulation or dissipation during the deformation of Dode-NTs.

Additionally, to investigate the hydrogenation process of Dode-NTs as a function of temperature and time, the Dode-NTs (15,0) and (0,11) nanotube configurations were selected. Both nanotubes were constructed with a length of 100 Å and similar diameters, in order to isolate the influence of chirality on the hydrogen adsorption process. The initial configurations of the gaseous hydrogen environment were generated using the Packmol software,^56^ and the adsorption simulations were performed over a total time of 250 ps within an NVT ensemble. Simulations were carried out at temperatures of 150, 300, 500, and 800 K.^41,57^ In these simulations, hydrogen is introduced as monatomic species to probe intrinsic chemisorption and site selectivity under an idealized flux of reactive *H*; explicit *H*_2_ dissociation and molecular physisorption are not captured and are left for future work.

## Results and Discussion

Figure 2a illustrates the variation of *E*_curv_ as a function of the chiral index *n* for Dode-NTs with (n,0) and (0,n) chiralities. As observed, *E*_curv_ decreases monotonically with increasing chiral index, which corresponds to an increase in the nanotube diameter. This trend reflects a lower surface stress required to bend larger-diameter nanotubes. Such behavior is consistent with previously reported trends for graphene and other nanotubes derived from novel 2D carbon allotropes, where the energetic cost of curvature is inversely proportional to the tube diameter.^6,58^

**Figure 2:**
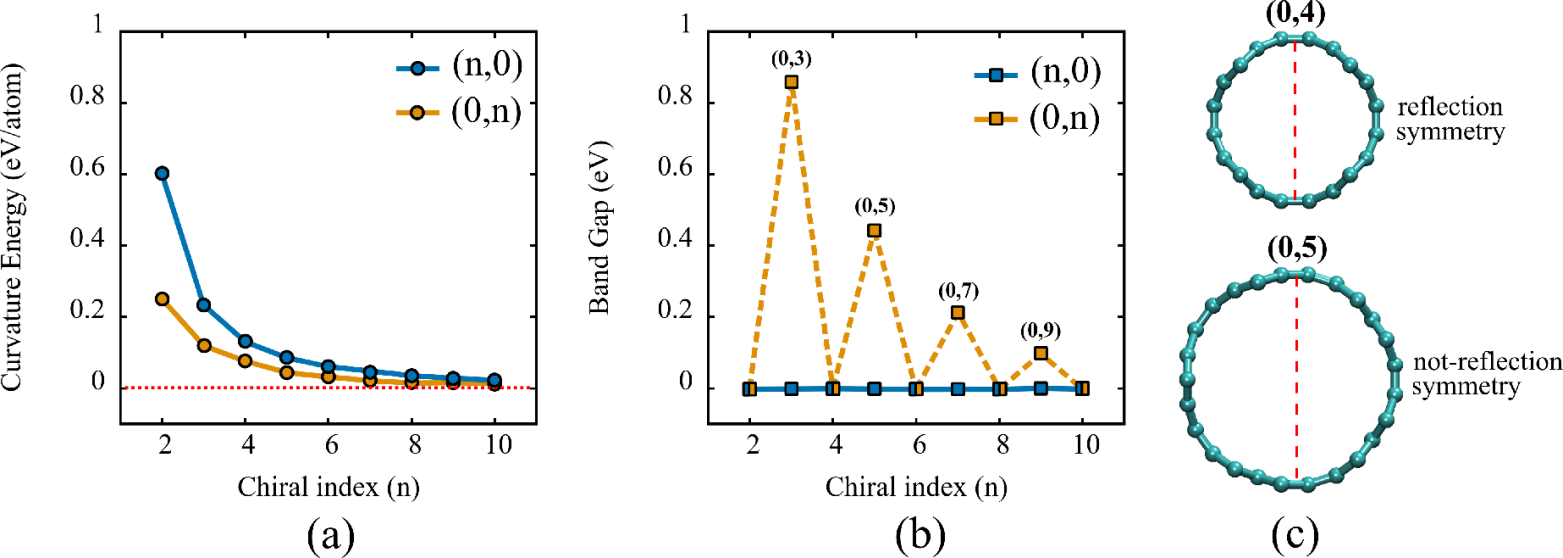
(a) Curvature energy as a function of the chiral index. (b) Band gap variation with respect to the chiral index. (c) Top view of Dode-NTs (0,4) and (0,5), showing that only even chiral indices preserve reflection symmetry.

It is noteworthy that the curvature energy is systematically higher for (n,0) nanotubes compared to their (0,n) counterparts at a similar chiral index. This asymmetry suggests a strong chirality-dependent effect on strain distribution during tube formation, likely resulting from the anisotropic arrangement of *C*_3_, *C*_6_, and *C*_12_ carbon rings in the dodecanophene lattice. The elevated *E*_curv_ values for (n,0) tubes may indicate the presence of unfavorable local distortions when bending occurs along specific pore orientations. This behavior has also been reported for TPDH-graphene nanotubes,^6^ where the (n,0) chirality exhibits a higher curvature energy. In contrast, for graphenylene nanotubes,^37^ no significant difference in curvature energy was observed among zigzag (n,0), armchair (n,n), and chiral (m,n) configurations. This discrepancy may be attributed to the higher symmetry in the distribution of carbon rings within the graphenylene structure, as compared to the structures of dodecanophene and TPDH-graphene, where the orientation of the carbon rings follows preferential directions.

In Figure 2b, the electronic band gap is shown as a function of the chiral index *n* for Dode-NTs with (n,0) and (0,n) configurations. All (n,0) nanotubes exhibit metallic behavior, with a zero band gap with bands crossing the Fermi level (see Figure S2). In contrast, (0,n) nanotubes display a more complex behavior: those with even values of *n* ( 2, 4, 6,···) maintain a metallic character, whereas tubes with odd values of *n* ( 3, 5, 7, ...) exhibit semiconducting behavior, with band gaps that decrease as the nanotube diameter increases. These oscillations in the band gap can be attributed to curvature-induced changes in orbital overlap and reflection symmetry breaking, which are more pronounced in narrower tubes (see Figure 2c).

Compared to other nanotubes derived from novel 2D carbon allotropes such as graphenylene,^37^ S-graphene,^59^ and HOT-graphene^36^ nanotubes which exhibit complex trends in band gap as a function of chirality, Dode-NTs with (0,n) chirality display an oscillatory behavior that follows a simple and predictable rule.

These findings underscore the structural stability and electronic robustness of Dode-NTs across various chiralities, reinforcing their potential as multifunctional materials for nanoelectronic applications, particularly in systems where consistent electronic properties under bending and deformation are required.

Figure 3 presents the Highest Occupied Crystal Orbital (HOCO) and the Lowest Unoccupied Crystal Orbital (LUCO) for Dode-NTs with (0,n) chirality and odd *n* values (3, 5, 7, 9). It should be noted that the HOCO and LUCO states correspond to the frontier orbitals at the valence band maximum (VBM) and conduction band minimum (CBM), respectively, thus providing the real-space distribution of these band-edge states.

**Figure 3:**
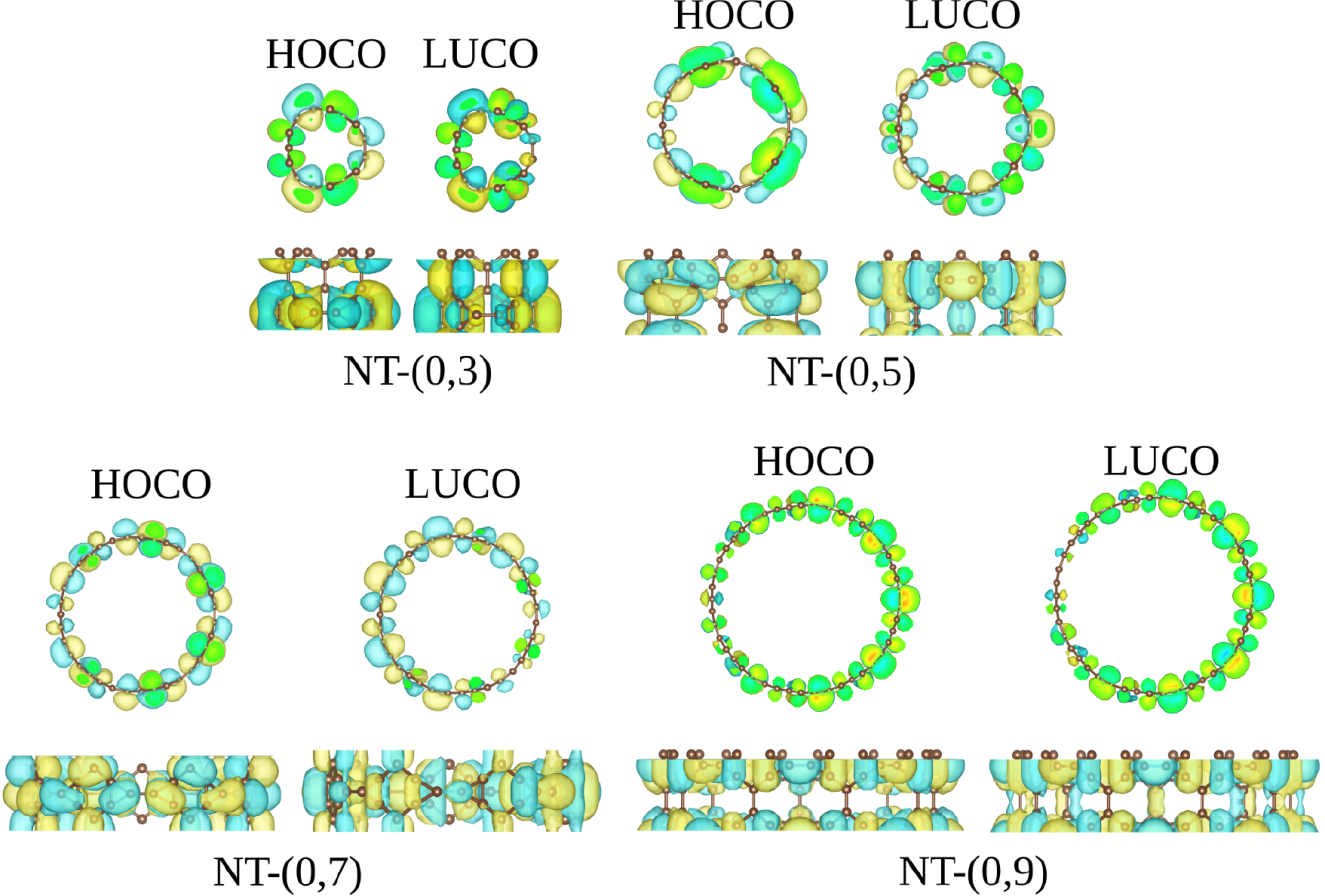
Highest Occupied Crystal Orbital (HOCO) and Lowest Unoccupied Crystal Orbital (LUCO) of Dode-NTs with (0,n) chirality with odd index.

As previously discussed in the electronic band structure analysis, these nanotubes exhibit a semiconducting character with a finite band gap. The spatial distribution of the orbitals reveals a clear distinction in both orientation and extent between the HOCO and LUCO states, which are critical for understanding their electronic behavior.

In all cases analyzed, the HOCO orbitals are oriented transversely with respect to the nanotube axis, i.e., they are arranged in planes perpendicular to the longitudinal direction (*z*-axis). This configuration indicates that the electrons in the HOCO state are primarily confined along the circumference of the nanotube, exhibiting limited delocalization along the z direction. Such a transverse localization hinders charge transport along the tube axis and is consistent with the presence of an energy gap.^6,60^

Conversely, the LUCO orbitals show a predominantly longitudinal orientation, aligned with the nanotube axis. This arrangement facilitates enhanced electronic connectivity along the tubular channel, allowing for more efficient charge carrier propagation upon excitation to the LUCO state.^6,60^ The distinct orbital orientations between HOCO and LUCO clearly indicate spatial separation of the frontier states, a characteristic feature of semiconducting systems.

Moreover, as the chiral index *n* increases, the orbitals tend to become more symmetric and delocalized, in agreement with the observed gradual narrowing of the band gap in larger-diameter nanotubes. This evolution suggests a smooth transition toward a more extended and stable electronic regime, further supporting the tunability of the material’s properties through geometric control.

Overall, the results in Figure 3 confirm that Dode-NTs with (0,n) chirality and odd *n* values exhibit an orbital organization that is fully consistent with their semiconducting nature. The transverse orientation of the HOCO and the longitudinal alignment of the LUCO provide a clear physical picture of the origin of the band gap and its potential modulation, positioning these nanotubes as promising candidates for directional electronic applications and optoelectronic devices with geometry-dependent functionalities.

Figure S3 displays the electronic band structures of Dode-NTs with (n,0) and (0,n) chiralities under uniaxial strain applied along the longitudinal direction of the nanotube. For each configuration, band structures are presented under approximately 3% and 5% strain, allowing for the assessment of how the electronic dispersion evolves under mechanical deformation.

In the case of the (n,0) nanotubes (top row), all configurations retain a metallic character even at 5% strain. No band gap opening is observed in any of the structures, indicating that deformation does not significantly alter the electronic nature of the system. This behavior is consistent with previous reports on the planar 2D phase of dodecanophene,^10^ which hosts a single Dirac cone that may shift slightly in momentum space under strain, but remains robust and undeformed even under substantial mechanical stress. The persistence of metallic behavior in (n,0) nanotubes suggests that the curvature introduced by rolling the 2D sheet in this direction does not appreciably disturb the electronic symmetry inherited from the 2D dodecanophene.

Similarly, for the (0,n) nanotubes (bottom row), the band dispersion near the Fermi level shows only minor changes in response to longitudinal deformation. The metallic or semiconducting character of these systems is preserved and remains dependent on the parity of the chiral *n* index, as previously discussed. These results highlight that both chiral families maintain their characteristic electronic responses under moderate strain, with the (0,n) series exhibiting band gap behavior that remains weakly sensitive to mechanical deformation while retaining its dependence on structural chirality.

Figure 4 presents the adsorption properties of a hydrogen atom on two types of Dode-NTs, namely (n,0) and (0,n). Panels (a) and (d) depict the three nonequivalent adsorption sites considered in the study, labeled as *S*_1_, *S*_2_, and *S*_3_. These sites were selected based on the local symmetry and atomic coordination within the dodecanophene lattice: *S*_1_ corresponds to a bond adjacent to a *C*_3_ ring, where bond angles induce higher local strain; *S*_2_ is located at the *C*_3_ - *C*_6_ interface, representing a mixed coordination environment; and *S*_3_ corresponds to a bond within a *C*_6_ ring, with a local symmetry closer to that of graphene. This selection provides a well-defined framework to assess how the topological anisotropy of Dode-NTs influences hydrogen adsorption.

**Figure 4:**
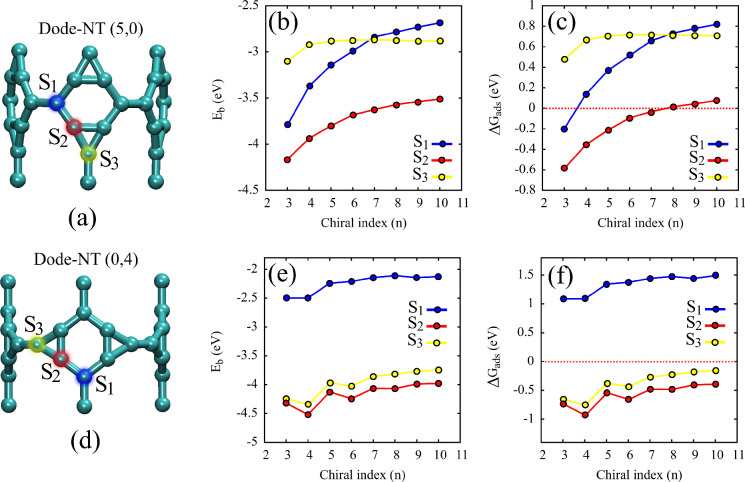
(a, d) Optimized atomic structures of Dode-NT (5,0) and Dode-NT (0,4), highlighting the three distinct hydrogen adsorption sites: *S*_1_, *S*_2_, and *S*_3_. (b, e) Binding energy (*E*_*b*_), and (c, f) adsorption free energy (Δ*G*_*ads*_) of a hydrogen atom as functions of the chiral index n, for each adsorption site. Panels (b, c) correspond to the Dode-NT (5,0), and panels (e,f) to the Dode-NT (0,4).

Figure S4 shows the optimized atomic structures of hydrogen adsorption at the three nonequivalent sites (*S*_1_, *S*_2_, and *S*_3_) for representative Dode-NTs. The relaxed geometries confirm that H atoms form covalent bonds with the carbon backbone, producing site-dependent orientations and local distortions. Together with the corresponding binding and free energies, these configurations provide direct evidence of how chirality and local topology govern hydrogen adsorption. After adsorption, the Dode-NTs retain their overall tubular geometry, with distortions confined to the vicinity of the adsorption sites. The *C*–*H* bond length remains within 1.10–1.12 Å, while neighboring *C*–*C* bonds exhibit only slight elongations (≤0.1 Å). These results indicate that hydrogen adsorption primarily induces local rehybridization effects without compromising the structural integrity of the nanotube framework.

Panels (b) and (e) show the *E*_b_, while panels (c) and (f) display the Δ*G*_ads_ as a function of the chiral index *n* for each site. These results enable a detailed evaluation of the thermodynamic favorability and highlight the influence of both chirality and curvature on hydrogen adsorption in Dode-NTs.

In both types of Dode-NTs, the *E*_b_ trends (panels (b) and (e)) reveal a clear energetic preference among the different adsorption sites, along with a noticeable effect of curvature. For the Dode-NTs (n,0), site *S*_2_ consistently exhibits the strongest hydrogen adsorption across all values of the chiral index *n*, with binding energies (*E*_*b*_) ranging approximately from −4.17 eV to −3.51 eV as *n* increases. Sites *S*_1_ and *S*_3_ show weaker interactions, with *S*_3_ displaying the least favorable adsorption in the range *n* = 3 to 6. However, for higher chiral indices (*n* = 7 to 10), site *S*_1_ becomes the least favorable.

In the case of Dode-NTs (0,n), site *S*_2_ also shows the strongest adsorption throughout the range of chiral indices, with *E*_*b*_ values between −4.31 eV and −3.98 eV. Site *S*_3_ exhibits slightly less negative binding energies, indicating a lower interaction. Site *S*_1_ consistently shows the weakest adsorption, suggesting a significantly lower C*–*H* interaction strength compared to sites *S*_2_ and *S*_3_.

Panels (c) and (f) of Figure 4 show how Δ*G*_ads_ varies as a function of the chiral index *n* for each of the three adsorption sites. For the Dode-NTs (n,0) (panel c), site *S*_2_ displays Δ*G*_ads_ values ranging from approximately −0.62 to 0.08 eV as *n* increases. This trend identifies *S*_2_ as the most favorable site for HER, particularly in larger-diameter nanotubes where the values approach the catalytic optimum. In contrast, sites *S*_1_ and *S*_3_ show more positive values, with *S*_1_ reaching up to 0.82 eV, indicating weak hydrogen adsorption and, consequently, low catalytic performance.

For the Dode-NTs (0,n) (panel f), the behavior is distinct. Sites *S*_2_ and *S*_3_ exhibit Δ*G*_ads_ values ranging from −0.69 eV to −0.12 eV, with minimal variation across different chiral indices. These values indicate stable adsorption, though slightly stronger than ideal in the case of *S*_3_. However, for *S*_3_ at larger diameters, the Δ*G*_ads_ approaches the optimal value for catalytic performance. Site *S*_2_ may still be considered catalytically active, particularly if electronic modifications such as doping or applied strain are employed to fine-tune the adsorption strength. In contrast, site *S*_1_ consistently shows Δ*G*_ads_ values above 1.0 eV, suggesting a thermodynamically unfavorable and catalytically inactive site.

As summarized in Table S1, we report local structural descriptors for representative Dode-NTs with (n,0) and (0,n) chiralities after *H* adsorption at the three adsorption sites. For the (n,0) tubes, *S*_2_ exhibits the largest local *sp* ^2^ → *sp* ^3^ conversion, as evidenced by a substantially larger planarity drop than *S*_3_ (≈61– 67° versus ≈48–50°) together with higher θ_*p*_ values. Concomitantly, the out-of-plane height *h*_*p*_ at *S*_2_ remains moderate (≈0.60–0.63 Å), indicating that stabilization of the C*–*H* bond arises primarily from angular changes (σ–π rehybridization) without incurring an excessive penalty in the surrounding σ network. This combination (effective rehybridization at limited geometric cost) rationalizes why *S*_2_ yields Δ*G*_ads_ values closest to the HER optimum across the (n,0) series, consistent with Figure 4.

Additionally, charge density difference maps were computed on the same real-space grid for the three sites (*S*_1_–*S*_3_) of representative Dode-NTs (5,0) and (0,4) (Figure S5). For both chiralities, *S*_2_ shows the most pronounced bond-aligned accumulation along C* - *H* together with depletion at the C*p_*z*_ lobe, consistent with enhanced local *sp* ^2^ → *sp* ^3^ rehybridization. These Δρ features correlate with the larger θ_*p*_ and planarity drops reported in Table S2 and rationalize the more favorable Δ*G*_ads_ at *S*_2_ relative to *S*_3_ and *S*_1_.

The orbital resolved PDOS of C* after *H* adsorption (Figure S6) provides a direct electronic signature of local *sp* ^2^ → *sp* ^3^ conversion. In metallic Dode-NT­(4,0), site *S*_2_ shows the strongest depletion of radial p weight (*p*_*x*_ + *p*_*y*_) at *E*_*f*_ together with enhanced bonding features at deeper energies (increased s + radial-p intensity below *E*_*f*_), consistent with a more pronounced σ–π rehybridization. By contrast, *S*_3_ exhibits only weak radial weight redistribution near *E*_*f*_, while *S*_1_ is intermediate. These trends mirror the larger planarity drops and higher _*p*_ quantified for *S*_2_ achieved at moderate *h*_*p*_ and rationalize why *S*_2_ yields Δ*G*_*ads*_ values closest to the HER optimum across the (n,0) series.

For Dode-NT­(0,4) the radial PDOS in the vicinity of *E*_*f*_ is overall smaller and shifted away from *E*_*f*_ ; nonetheless, *S*_2_ retains the clearest depletion pattern and the most evident bonding features, consistent with its comparatively stronger *C* – *H* bonding than *S*_1_/*S*_3_ and with the more moderate, band-alignment-sensitive activity of this family. In semiconducting Dode-NT­(0,5), *E*_*f*_ falls within a region of very low radial PDOS and the dominant *p*_*x*_ + *p*_*y*_ features accumulate at the band edges; *S*_2_ displays the radial states closest to *E*_*f*_, explaining its relative preference and its sensitivity to potential. These PDOS observations are fully consistent with the charge-density-difference maps (bond-aligned accumulation along C*–*H* and depletion at the C*–*p* lobe) and with the geometric descriptors reported in Tables S1–S2, thereby establishing the explicit link between local electronic structure, pyramidalization, and the site-selective HER thermodynamics.

Given that Δ*G*_ads_ is sensitive to curvature and local bond angles, axial strain is expected to modulate hydrogen adsorption thermodynamics and site selectivity; likewise, structural defects introduce localized electronic states and under-coordinated sites that can strengthen *H* binding (i.e., render Δ*G*_ads_ more negative) and modify selectivity and kinetics. In the same vein, point vacancies and B/N dopants are expected to create localized states near *E*_*f*_, shift the Fermi level (p-type for B, n-type for N), and renormalize the semiconducting gaps of (0,n) tubes, thereby shifting Δ*G*_*ads*_ and site preferences at defect/dopant locations. A more rigorous quantitative assessment of these effects will require dispersion-corrected DFT under finite strain with systematic configurational sampling; such analyses are beyond the scope of the present study and will be addressed in future work.

In the literature, platinum (Pt) serves as the HER benchmark, sitting near the top of the volcano with a nearly thermo-neutral Δ*G*_*ads*_ ≈ 0 eV. To position Dode-NTs within the broader landscape, we note that contemporary metal-free carbons span a wide activity window: pristine graphene typically binds H too weakly, whereas curvature, heteroatom doping (e.g., N, B),^61^ and nonbenzenoid ring motifs can tune Δ*G*_ads_ toward thermo-neutrality.^62^ In this context, the Δ*G*_ads_ values we obtain for Dode-NTs, especially at site *S*_2_ in the (n,0) family and for larger diameters fall in the same favorable range commonly associated with state-of-the-art metal-free carbons and close to Pt, underscoring that structural anisotropy and curvature in Dode-NTs provide an intrinsic, dopant-free route to HER relevant thermodynamics.

While Δ*G*_ads_ is a direct descriptor of HER activity, it does not quantify hydrogen storage capacity. A rigorous assessment of storage will require explicit simulations of molecular *H*_2_ physisorption on Dode-NTs, incorporating dispersion-corrected energetics and systematic configurational sampling, which are beyond the scope of the present study.

Figure 5 presents the behavior of the Young’s modulus as a function of the nanotube radius for Dode-NTs with (n,0) and (0,n) chiralities, calculated using DFT. The Young’s modulus provides a measure of the elastic stiffness of a material under uniaxial deformation and, in the case of nanotubes, reflects both the mechanical resistance of the atomic structure and the effects of curvature and chirality.

**Figure 5:**
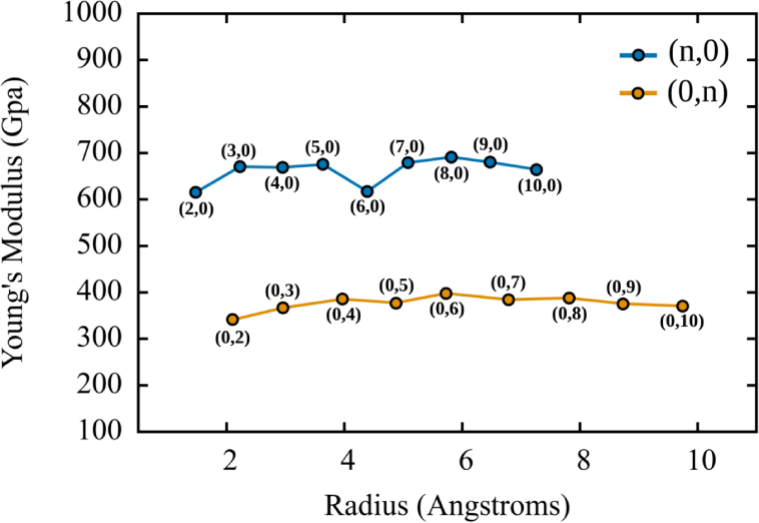
(a) Young’s modulus as a function of the nanotube radius for Dode-NTs with (n,0) and (0,n) chiralities, calculated via DFT.

The results reveal a clear distinction between the two families of nanotubes analyzed. The Dode-NTs (n,0) exhibit significantly higher Young’s modulus values, ranging approximately from 614 to 692 GPa. This high stiffness indicates strong mechanical resistance to deformation, likely attributed to a more efficient alignment of covalent bonds along the nanotube axis. Furthermore, the variation with radius does not follow a strictly monotonic trend, suggesting structural modulation influenced by resonance effects and curvature as a function of the chiral index.^34^

In contrast, the Dode-NTs (0,n) display substantially lower Young’s modulus values, ranging from 342 to 398 GPa. This reduction can be attributed to the different bond orientation in this configuration, where the dodecanophene rings are not optimally aligned along the tube axis, thereby reducing the ability to withstand longitudinal tensile stress. In this case, the stiffness values tend to stabilize as the radius increases, indicating a weaker dependence on curvature.

Overall, these results highlight the mechanical anisotropy of Dode-NTs and confirm that their elastic properties are strongly influenced by chirality and only moderately affected by curvature, as already reported for nanotube structures formed by 2D carbon allotropes, which already present anisotropy in their elastic properties.^2,6,8^ The higher rigidity observed in the Dode-NTs (n,0) positions them as more robust candidates for applications requiring structural integrity under mechanical stress, whereas the Dode-NTs (0,n) may offer advantages in applications that demand greater flexibility or deformation tolerance. For completeness, we also evaluated the axial Young’s modulus of Dode-NT­(10,0) including dispersion corrections using two vdW schemes (KLMLL and KBM) obtaining 628.223 ± 8.501 GPa and 634.854 ± 6.711 GPa, respectively, compared to 664.543 ± 11.450 GPa without vdW. These results indicate that vdW interactions lead to a modest (∼4–6%) reduction in the absolute stiffness, while preserving the overall magnitude and hierarchy of the elastic response discussed above.

Figure 6 presents the mechanical properties of Dode-NTs at room temperature, calculated via MD simulations. Specifically, panel (a) shows the Young’s modulus as a function of the nanotube radius, while panel (b) displays the ultimate tensile strength (UTS), both evaluated for a series of (n,0) and (0,n) chiralities. Here we restrict the MD-based mechanical tests to *T* = 300 K, representative of ambient conditions; a systematic investigation of the temperature dependence of Young’s modulus and UTS over a broader range of *T* and chiralities would require an extensive additional set of large-scale simulations and is therefore left for future work.

**Figure 6:**
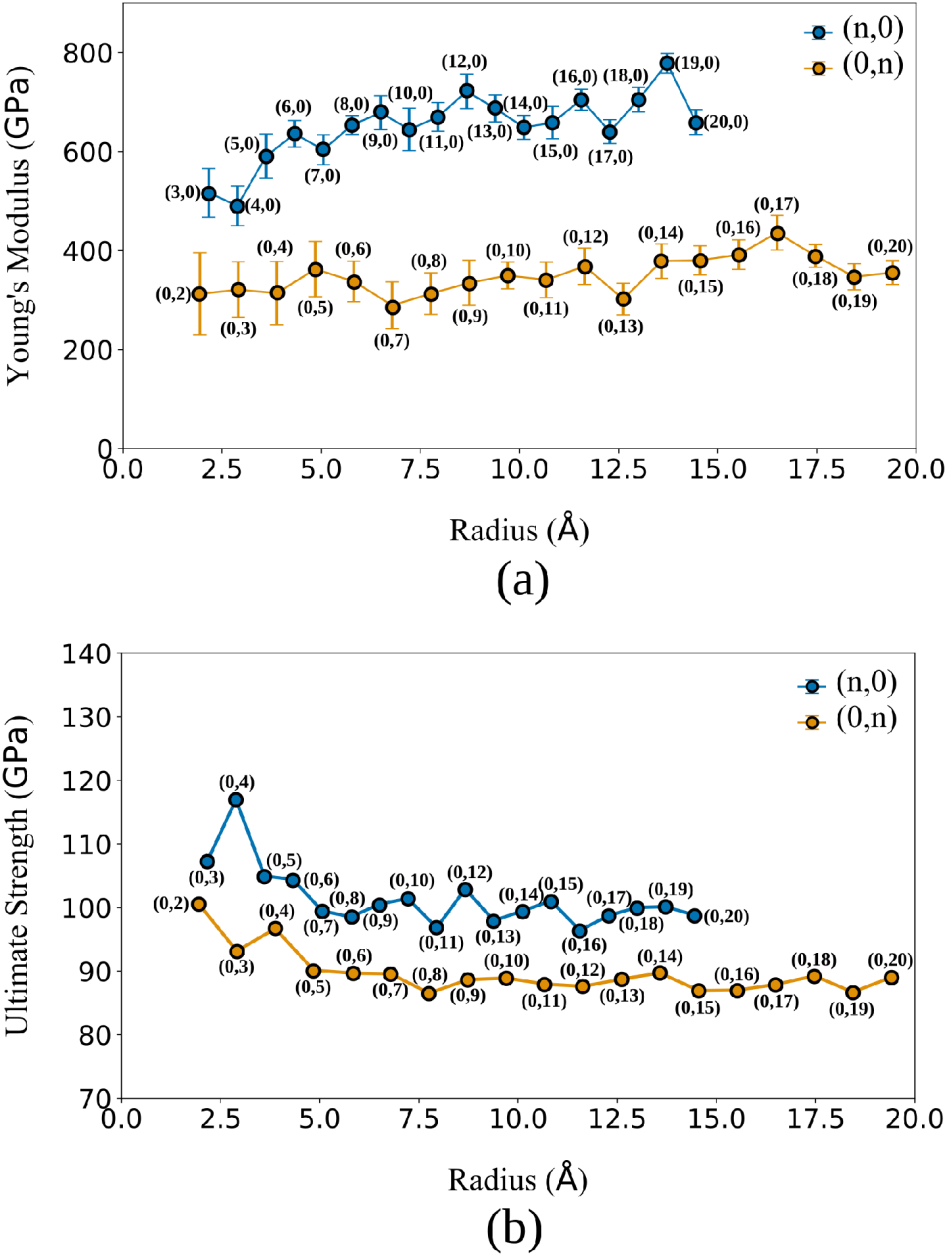
(a) Young’s modulus and (b) ultimate tensile strength as functions of the nanotube radius for Dode-NTs with (n,0) and (0,n) chiralities, calculated via MD simulations.

The results in panel (a) confirm the trends previously observed from DFT calculations, showing a clear mechanical anisotropy between the two types of Dode-NTs. Additionally, the thermal stability of the Dode-NTs at room temperature was confirmed. Throughout the trajectories, the structures maintained their integrity without radial collapse or premature bond cleavage under equilibrium conditions.

The Dode-NTs (n,0) exhibit significantly higher Young’s modulus values, ranging from approximately 500 to 750 GPa, with a tendency to increase with radius. In contrast, the Dode-NTs (0,n) display much lower Young’s modulus values, typically between 300 and 430 GPa, with a more moderate radius dependence. These findings indicate that the (n,0) configuration possesses a more robust atomic structure aligned along the tube axis, which enhances its resistance to uniaxial deformation.^2,6,8,9^

Panel (b) shows the ultimate tensile strength for both types of Dode-NTs. Again, the Dode-NTs (n,0) outperform their Dode-NTs (0,n) counterparts, exhibiting UTS values in the range of 100–120 GPa, with a relatively stable trend across the radius. On the other hand, the (0,n) nanotubes show lower UTS values, between 80 and 100 GPa, with a slightly decreasing trend as the radius increases. These differences can be attributed to the directionality of the covalent bonds within the dodecanophene structure, which in the (n,0) configuration are better oriented to resist tensile forces.^2,6,8,9^

Figure 7 provides further insights into the mechanical behavior of Dode-NTs under uniaxial tensile deformation. Panel (a) displays the stress–strain curves for dodecanophene-NT­(15,0) and (0,11), while panels (b) and (c) illustrate the corresponding atomic configurations and von Mises stress distributions at various strain levels.

**Figure 7:**
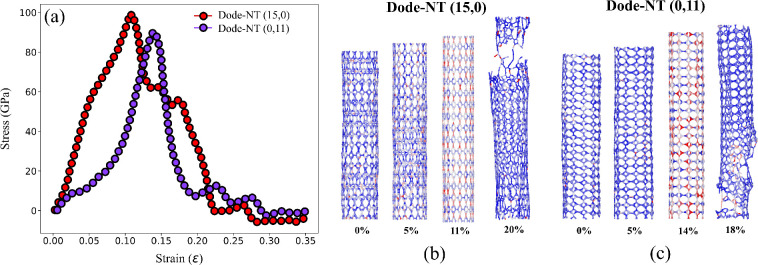
(a) Stress–strain curves for Dode-NTs (15,0) and (0,11). (b, c) Evolution of the von Mises stress under increasing strain for Dode-NTs (15,0) and (0,11).

From panel (a), the Dode-NT (15,0) exhibits a steeper stress–strain slope in the elastic regime, confirming its higher Young’s modulus compared to Dode-NT (0,11). The Dode-NT (15,0) also reaches a higher ultimate tensile strength (∼106 GPa) and fails at a strain of approximately 0.20. In contrast, Dode-NT (0,11) fails at a lower peak stress (∼95 GPa) and at a smaller strain (∼0.18), indicating that the (0,n) configuration is mechanically less stiff and less strong than its (n,0) counterpart.

Panels (b) and (c) show the evolution of the von Mises stress distribution throughout the deformation process. For Dode-NT (15,0), stress concentration begins to develop around 11% strain, with visible localization patterns indicating stress accumulation primarily within the *C*_3_ carbon rings.^1,6,63^ At 20% strain, the fracture threshold is reached, and structural failure becomes evident. In contrast, Dode-NT (0,11) displays a more homogeneous stress distribution during deformation, with moderate stress localization observed near the *C*_6_ carbon rings at 14% strain. By 18% strain, fracture is clearly observed, marked by the tearing of the nanotube structure.

These observations reinforce the mechanical anisotropy of Dode-NTs, where Dode-NTs with (n,0) chirality demonstrate superior stiffness and strength, while Dode-NTs (0,n) exhibit greater deformation tolerance. This distinction is critical when selecting optimal chiralities for applications that demand either mechanical robustness or enhanced flexibility.

Figure 8 provides an atomistic-level analysis of the structural response of Dode-NTs under uniaxial tensile deformation, by tracking the evolution of bond lengths and bond angles, as defined previously in Figure 1c,d. Panels (a) and (b) show the evolution of ten representative *C*–*C* bond lengths as a function of strain for Dode-NT (15,0) and Dode-NT (0,11), respectively, while panels (c) and (d) present the angular distribution of eight selected bond angles during the stretching process.^3,6,64–66^

**Figure 8:**
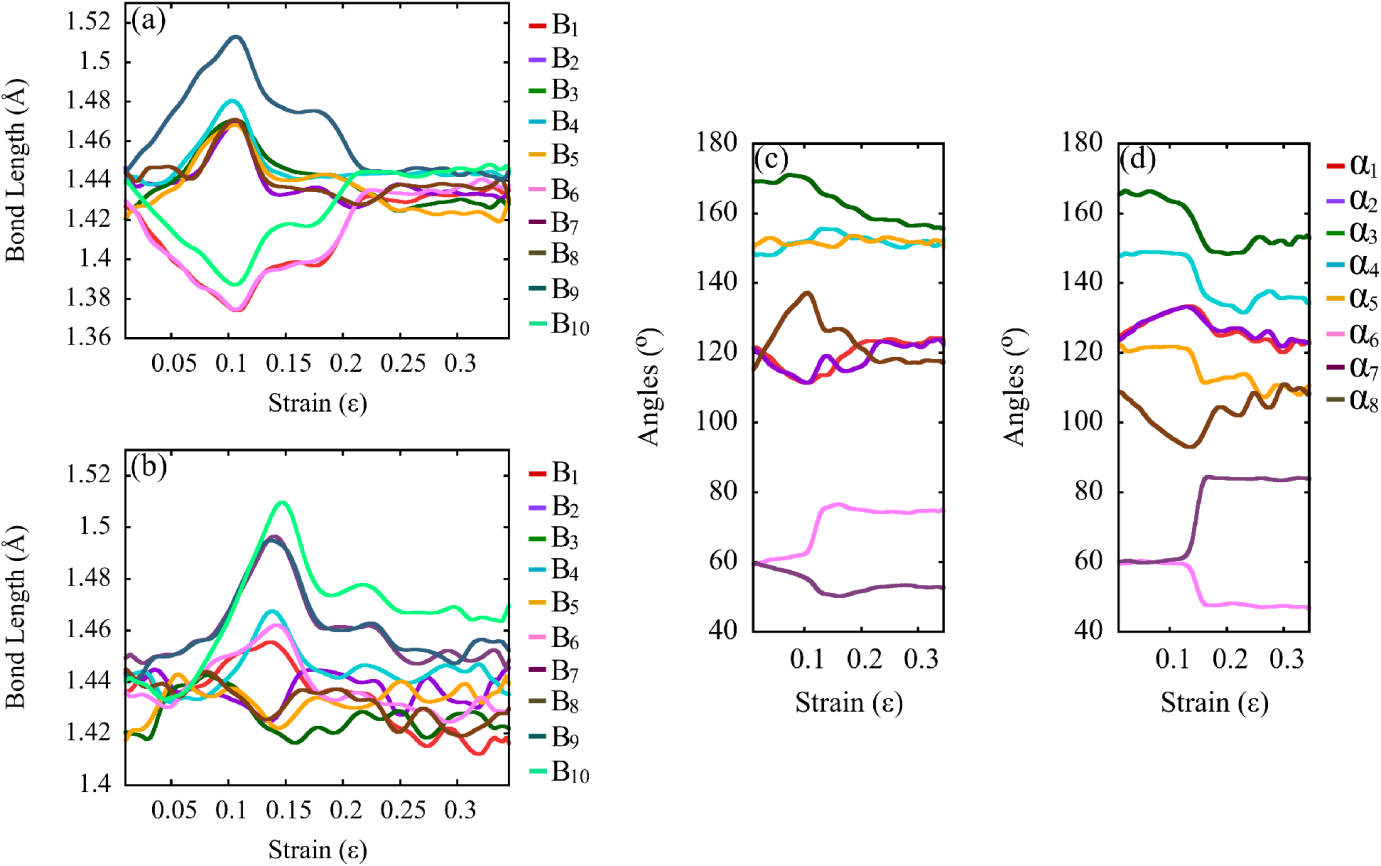
(a, b) Evolution of bond length with strain for Dode-NTs (15,0) and (0,11), respectively. (c, d) Angular distribution as a function of strain for Dode-NTs (15,0) and (0,11), respectively.

In panel (a), the Dode-NT (15,0) configuration exhibits significant fluctuations in all bond lengths, reaching local maxima and minima between approximately 8% and 10% strain, followed by a sharp drop after 12% strain, marking the onset of structural failure. The variation in bond length reaches up to ∼0.15 Å, indicating localized stress accumulation and weakening of bonds prior to fracture.^6,64,66^ This behavior is consistent with the stress localization observed in the von Mises stress plots (see Figure 7b). In contrast, panel (b), corresponding to Dode-NT (0,11), reveals only modest increases in bond lengths, with a maximum elongation of ∼0.06 Å. This moderate variation supports the notion of a more distributed and progressive deformation mechanism, aligned with the greater ductility observed for Dode-NT (0,11).^6,64,66^

Panels (c) and (d) show the evolution of bond angles under increasing strain. For Dode-NT (15,0), several angles display pronounced variations in the critical strain region (8–10%), especially angle α_8_, which undergoes a sharp increase, indicating localized structural distortion prior to fracture.^6,64,66^ Significant deviations are also observed in α_1_ and α_2_, reflecting bond rotations and angular openings associated with structural collapse.

On the other hand, for Dode-NT (0,11), shown in panel (d), the angular evolution is more gradual, without abrupt transitions.^6,64,66^ Although certain angles such as α_8_ also exhibit notable deviations under tension, these changes are smoother and less extreme than in Dode-NT (15,0), further supporting a more uniform stress distribution and a delayed onset of complete fracture.

In general, we identify *C*_3_-rich motifs and *C*_3_–*C*_6_ junctions aligned with the deformation as the weakest link sites where the cracks preferentially nucleate. In contrast, *C*_12_ rings are not initiation foci; they buffer strain through angle bending and ovalization. This distinction explains the more brittle response of Dode-NTs (n,0), dominated by *C*_3_ motifs, versus the more ductile behavior of (0,n), where *C*_12_ helps redistribute stress. From a design point of view, reducing contiguous *C*_3_ chains and increasing the presence of *C*_12_ rings or disrupting *C*_3_–*C*_6_ junctions can increase fracture deformation and improve toughness.

Taken together, these results provide atomistic evidence of the mechanical anisotropy between Dode-NTs (n,0) and (0,n). The (n,0) configuration undergoes highly localized bond elongations and angular distortions before fracturing, characteristic of brittle behavior, while its (0,n) counterpart deforms more uniformly, allowing for greater strain tolerance and a more ductile mechanical response.

Figure 9 presents a statistical study of hydrogen atom adsorption on Dode-NTs with (15,0) and (0,11) chiralities in a monatomic hydrogen environment.^41,57^ Panels (a) and (b) display the initial atomic configurations of both nanotubes placed within a periodic simulation cell filled with hydrogen atoms, used to emulate the adsorption environment. The lower panels (c) and (d) illustrate the temporal evolution of the percentage of occupied adsorption sites defined as the formation of *C* –*H* bonds as a function of temperature and time, for the three distinct (nonequivalent) adsorption sites previously introduced (see Figure 4a,d).

**Figure 9:**
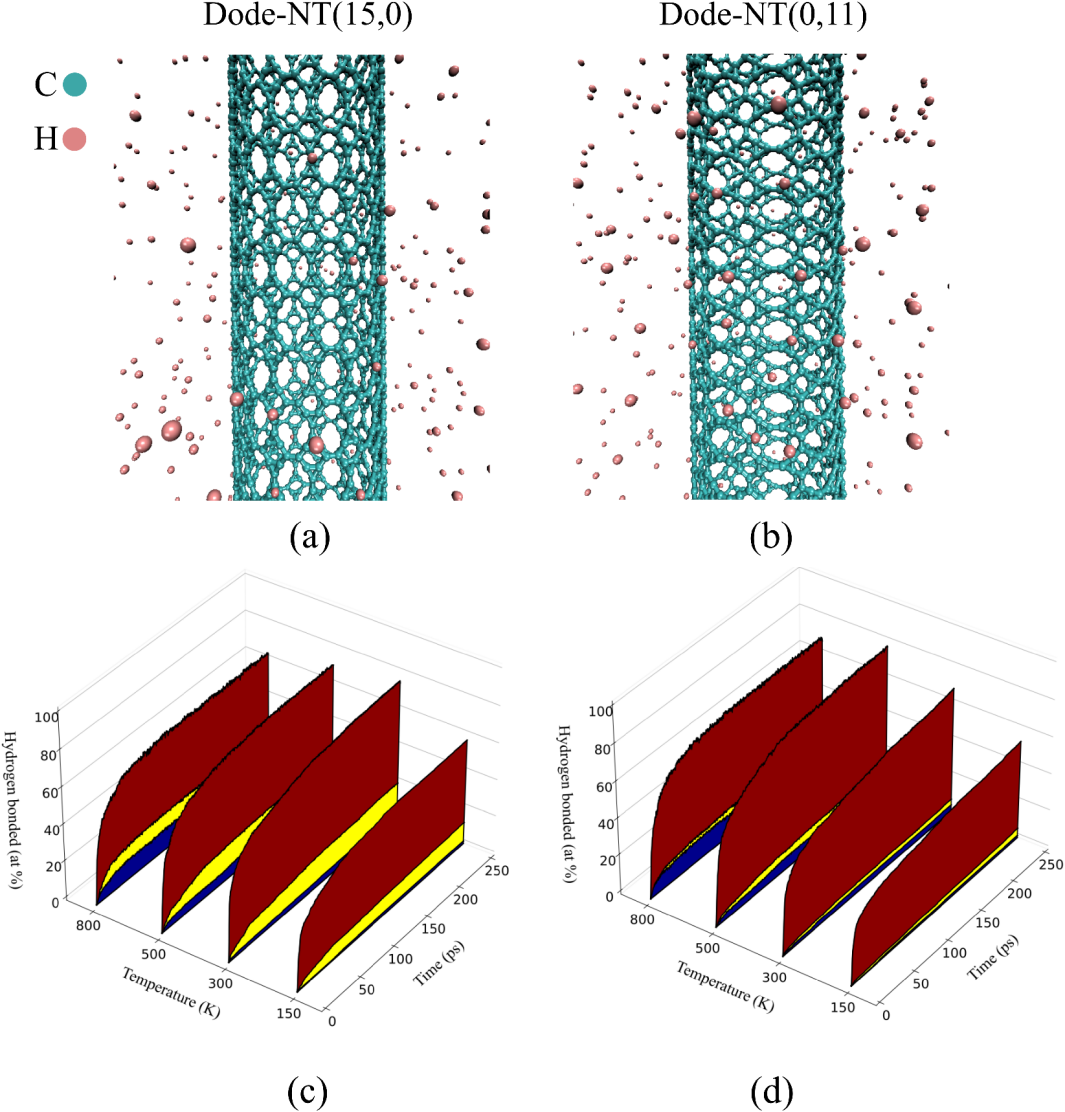
(a, b) Atomic structures of Dode-NTs (15,0) and (0,11) in a monatomic hydrogen environment. (c, d) Statistical analysis of *C*–*H* bond formation across three distinct (nonequivalent) adsorption sites (see Figure 4a for the color legend) for Dode-NTs (15,0) and (0,11).

In both cases, a progressive increase in the number of *C* –*H* bonds is observed with time and rising temperature, consistent with a thermodynamically favorable adsorption process.^41,57^ However, notable differences arise between the (n,0) and (0,n) configurations.

For the Dode-NT (15,0) system, shown in panel (c), the occupation rate of adsorption sites is faster, and the occupation is more pronounced, particularly at temperatures exceeding 600 K. This behavior indicates a higher surface reactivity for this chirality, in agreement with the previously discussed near-zero adsorption free energy (Δ*G*_*ads*_) values for site *S*_2_. Moreover, a clear differentiation is observed among the three nonequivalent adsorption sites, both in the rate and extent of adsorption, suggesting a strong dependence on the local orientation of the *C* –*H* bond relative to the nanotube geometry.

In contrast, for the Dode-NT (0,11) system, shown in panel (d), the fraction of occupied sites increases more gradually, and the distinction among the three adsorption sites is less pronounced. This reflects a lower overall reactivity and a reduced sensitivity to structural variation between sites, consistent with the smaller dispersion in Δ*G*_*ads*_ values reported for this family of nanotubes.

Overall, the results reinforce the conclusion that both chirality and local geometry play a critical role in governing hydrogen chemisorption on Dode-NTs. The Dode-NT (15,0) exhibits enhanced functionalization efficiency under elevated thermal conditions, making it promising candidates for catalytic or hydrogen storage applications where high surface reactivity is desired. In contrast, the Dode-NT (0,11) offer a more gradual and uniform functionalization profile, which may be advantageous in applications requiring controlled adsorption dynamics.

## Conclusions

This work presents a theoretical investigation of the electronic, mechanical, and hydrogen adsorption properties of Dode-NTs with (n,0) and (0,n) chiralities, based on DFT calculations and reactive classical MD simulations.

As summarized in Table S2, Dode-NTs (n,0) exhibit a robust metallic character even under uniaxial strain, whereas (0,n) nanotubes with odd chiral indices show semiconducting behavior with spatially separated HOCO and LUCO orbitals. These semiconducting features are geometry-dependent, offering tunability through structural design. At the microscopic level, this contrast originates from the way curvature and the anisotropic arrangement of *C*_3_, *C*_6_, and *C*_12_ rings break reflection symmetry in the (0,n) family while preserving the Dirac-like metallic manifold inherited from the 2D sheet in the (n,0) tubes. The overall stability of the electronic behavior under mechanical deformation positions these nanotubes as promising candidates for flexible electronic and optoelectronic devices operating under variable conditions.

Regarding hydrogen adsorption, both thermodynamic and statistical analyses indicate that site *S*_2_ in Dode-NT (n,0) particularly for larger diameters displays adsorption free energy (Δ*G*_*ads*_) values close to the catalytic optimum for the hydrogen evolution reaction. In contrast, Dode-NTs (0,n) exhibit weaker sensitivity to chirality, with sites *S*_2_ and *S*_3_ being moderately active yet less efficient for smaller diameters. These results highlight the critical role of local bonding orientation and curvature in surface reactivity, establishing (n,0) nanotubes as promising platforms for electrochemical catalysis or hydrogen storage applications. This behavior can be traced back to stronger local pyramidalization and σ–π rehybridization at *C*_3_-rich *C*_3_–*C*_6_ junctions aligned with the tube axis in the (n,0) family, which stabilize the C*–*H* bond at moderate geometric cost, whereas the larger fraction of *C*_12_ rings in (0,n) tubes redistributes strain and weakens the sensitivity of adsorption to chirality.

However, quantitative hydrogen storage metrics will require explicit *H*_2_ physisorption simulations with dispersion-corrected DFT and systematic configurational sampling, which we leave for future work. Likewise, a more realistic description of hydrogenation dynamics would require explicit modeling of *H*_2_ dissociation in the MD simulations, beyond the idealized monatomic hydrogen environment considered here. Moreover, a full kinetic and coverage-dependent description of HER on Dode-NTs, including activation barriers for Tafel and Heyrovsky steps, higher surface coverages, and the influence of axial strain, defects and heteroatom doping on Δ*G*_*ads*_, will require dedicated constant potential NEB/CI-NEB studies with explicit solvent and extensive configurational sampling, and is also left for future work.

In terms of mechanical performance, both DFT and MD confirm that Dode-NTs (n,0) possess higher stiffness and tensile strength, while Dode-NTs (0,n) nanotubes exhibit a more ductile and homogeneous deformation response. This anisotropy is further supported by atomistic analysis of bond and angle evolution under strain, where fracture mechanisms are found to be localized in Dode-NTs (n,0) and more evenly distributed in Dode-NTs (0,n). At the microscopic level, the stiffer and more brittle response of the (n,0) tubes is linked to chains of *C*_3_ rings and *C*_3_–*C*_6_ junctions aligned with the loading direction, which act as preferential crack-initiation motifs, whereas the presence of larger *C*_12_ rings in (0,n) tubes allows strain to be redistributed through angle bending and pore ovalization, leading to a more ductile behavior.

While the present mechanical analysis is limited to uniaxial tensile loading of pristine Dode-NTs at *T* = 300 K, previous work on the dodecanophene sheet has shown that temperature, defects, and stacking can further modulate the elastic response. Extending the present study to other loading modes, finite-temperature trends, and defected or doped Dode-NTs thus constitutes a natural next step toward a comprehensive mechanical characterization.

Finally, dynamic adsorption simulations in hydrogen-rich environments confirm a higher degree of surface functionalization for Dode-NTs (n,0) nanotubes, reinforcing their suitability for applications requiring elevated thermal reactivity. This enhanced functionalization efficiency reflects the higher density and accessibility of intrinsically active *C*_3_-based sites in the (n,0) family, consistent with the thermodynamic and geometric trends discussed above. Experimentally, our predictions can be tested via STM/STS^67^ mapping of frontier-state localization in semiconducting (0,n) tubes, polarized-Raman together with nanoindentation or in situ tensile tests to quantify mechanical anisotropy and failure modes,^68,69^ and EC-STM/XPS or TPD after controlled H dosing to assess site-specific chemisorption and thermodynamics relevant to HER.^70–72^

Overall, this study demonstrates that Dode-NTs constitute a versatile class of nanomaterials with highly tunable properties through structural engineering. Among them, Dode-NTs (n,0) configurations emerge as particularly promising for applications in catalysis, energy storage, nanoelectronics, and high-performance structural components. Looking ahead, we will extend these trends to armchair (n,n) and truly chiral (n,m) Dode-NTs whose longer periodicity and many nonequivalent adsorption sites expand the configurational space and enable a complete structure–property map.

## Supplementary Material



## Notes

Stress–strain curves (Figure S1); electronic band structures and strain effects (Figures S2–S3); H-adsorption geometries/band structures with *E*_*b*_ and Δ*G*_ads_ (Figure S4); charge density difference isosurfaces (Figure S5); orbital-resolved PDOS after H adsorption (Figure S6); local geometric descriptors (Table S1); and summary of main properties by chirality (Table S2) (PDF)
