# Supplementary material for: Chirality-Driven Electronic, Mechanical, and Hydrogen Adsorption Properties of Dodecanophene Nanotubes
Source: ACS Omega. 2026 Jan 22;11(4):5211–26. doi: 10.1021/acsomega.5c07529 (PMC12878782; doi:10.1021/acsomega.5c07529)
Supplement: Supplementary file 1 [file ao5c07529_si_002.pdf]

Chirality-Driven Electronic, Mechanical, and Hydrogen Adsorption Properties of Dodecanophene Nanotubes

Juan Rafael Gomez Quispe,[†] Fernando Guido Ordinola Sanchez,[‡] R. M. Guzmán-Arellano,[‡] Chachi Rojas-Ayala,[‡] and Pedro Alves da Silva Autreto[*,†]

†*Center of Natural and Human Sciences, Federal University of ABC, Santo Andre, Sao Paulo, Brazil*

‡*Facultad de Ciencias Físicas, Universidad Nacional Mayor de San Marcos, 15081 Lima, Peru*

E-mail: pedro.autreto@ufabc.edu.br

Phone: +55 (19) 98279-4988

Supporting Information Available

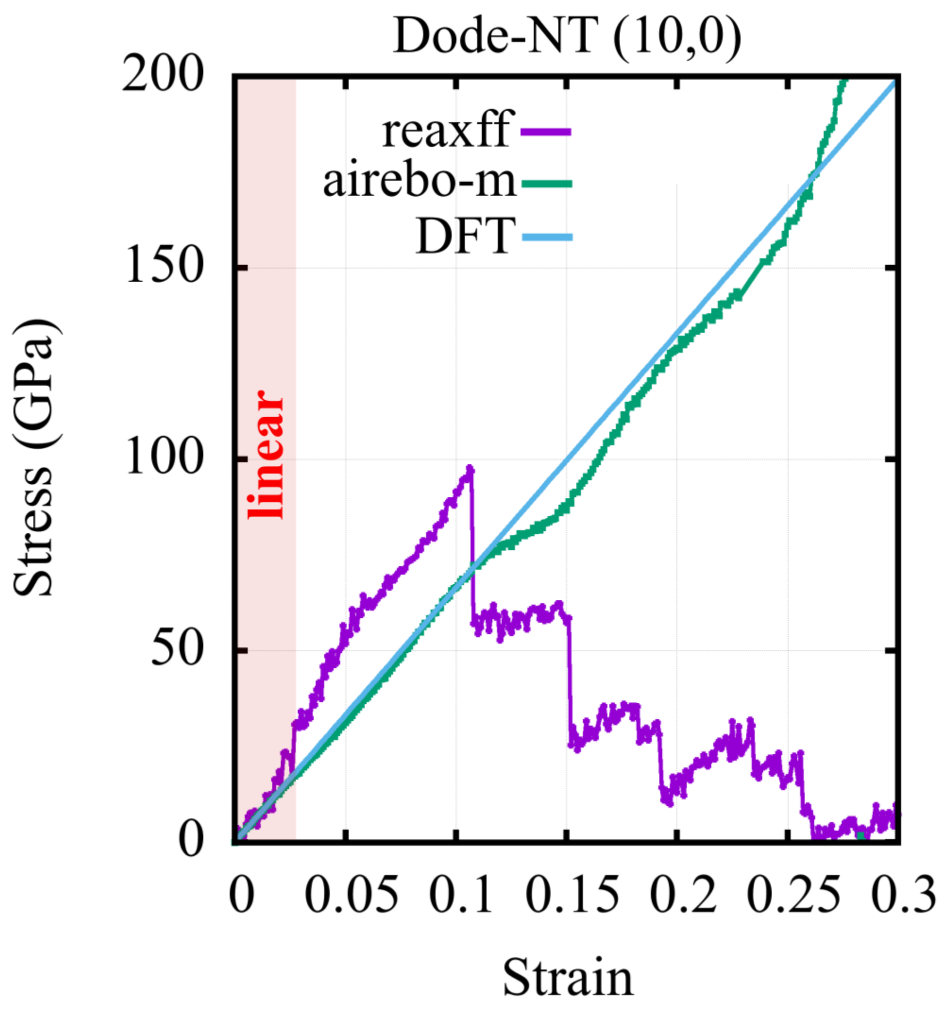

Figure S1: Stress-strain curves for Dode-NT(10,0). The shaded area highlights the linear regime where AIREBO-m and ReaxFF match the DFT slope.

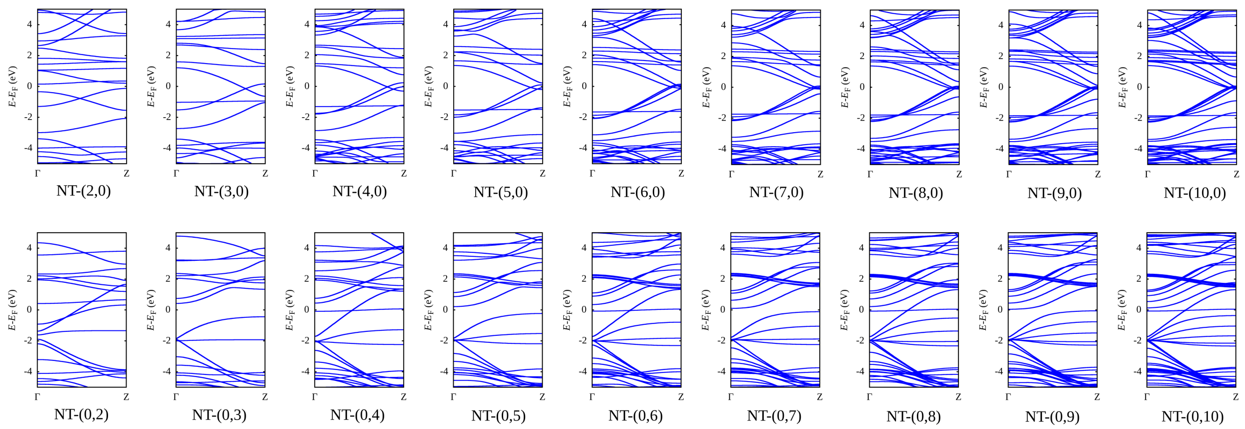

Figure S2: Electronic band structure of Dode-NTs with (n,0) and (0,n) chiralities.

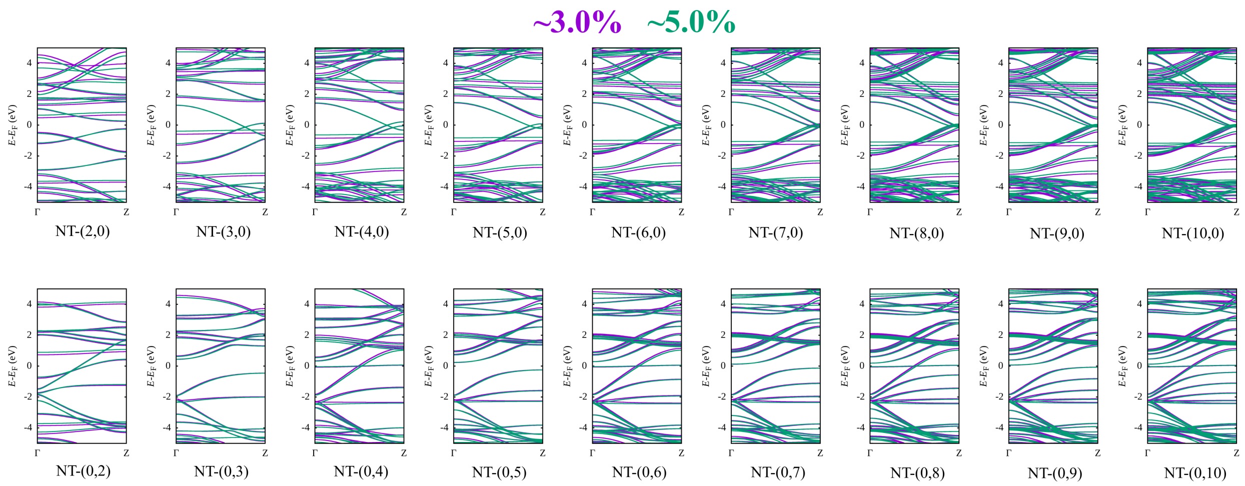

Figure S3: Electronic band structure of Dode-NTs with (n,0) and (0,n) chiralities under uniaxial strain.

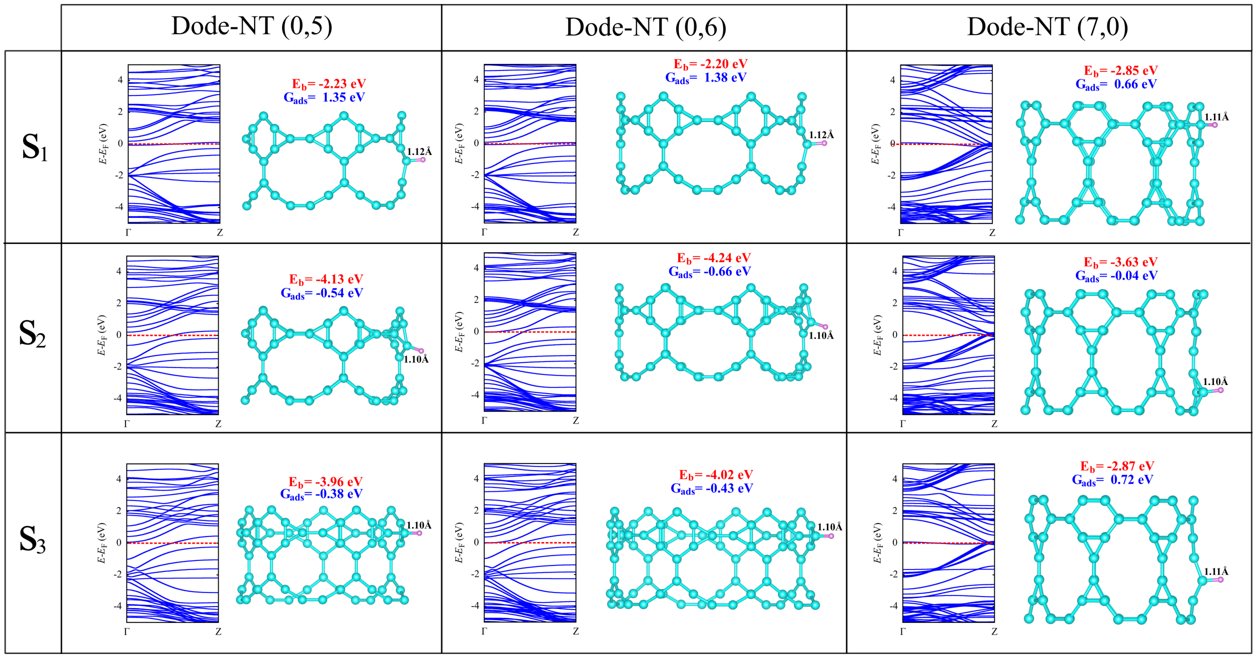

Figure S4. Optimized atomic structures of hydrogen adsorption at the three nonequivalent sites (S1, S2, and S3) for representative Dode-NTs: (0,5), (0,6), and (7,0). For each case, the electronic band structure is shown alongside the relaxed geometry after H adsorption. The calculated binding energies (E_b) and adsorption free energies (ΔG_{ads}) are indicated for each configuration. These results highlight how local site environment and nanotube chirality influence both the thermodynamics and the electronic response of hydrogen adsorption.

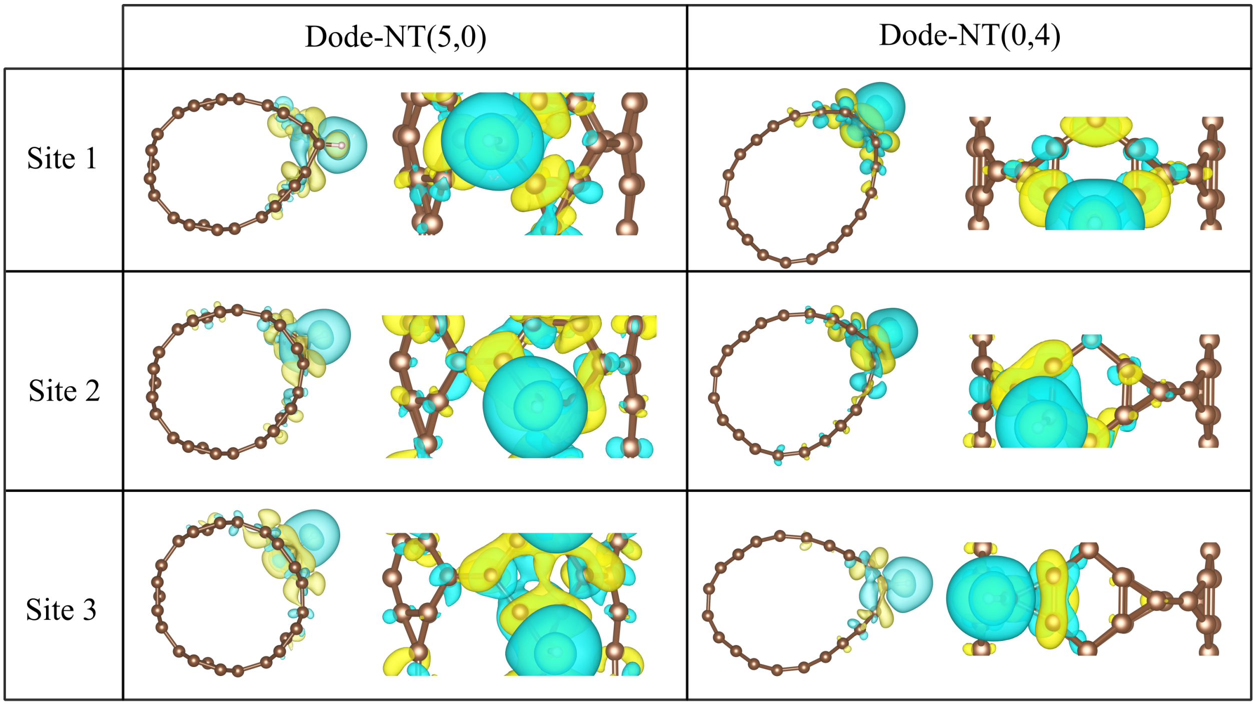

Figure S5. Charge density difference iso-surfaces for H adsorption at sites $S_1 - S_3$ on Dode-NT(5,0) and Dode-NT(0,4). The maps are computed as $\Delta\rho(r) = \rho(NT+H) - \rho(NT) - \rho(H)$ on the same real-space grid; positive (yellow) denotes charge accumulation and negative (blue/cyan) denotes depletion. Iso-values are symmetric and kept identical across all panels. Two views are shown for each case (left: ring view; right: side view). For both chiralities, site S_2 exhibits the most pronounced bond aligned accumulation along $C^* - H$ accompanied by depletion at the C^* pz lobe, indicating enhanced local $sp^2 \rightarrow sp^3$ re-hybridization and consistent with the more favorable ΔG_{ads} reported in the main text.

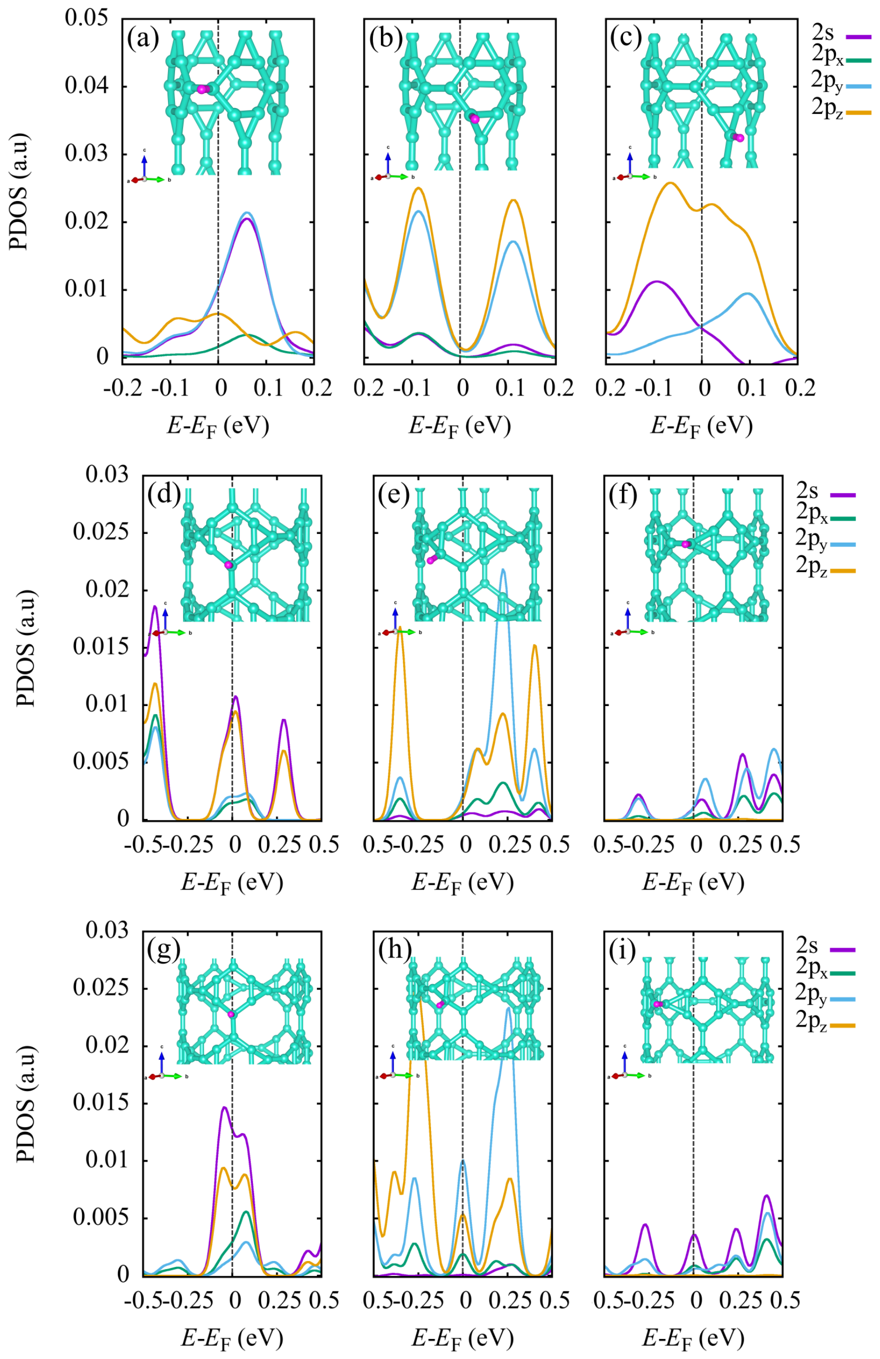

Figure S6. Orbital-resolved projected density of states (PDOS) on the adsorbing carbon atom C^{*} after H adsorption for: (a–c) Dode-NT(4,0), (d–f) Dode-NT(0,4), and (g–i) Dode-NT(0,5). Within each row: (a,d,g) site S_1; (b,e,h) site S_2; (c,f,i) site S_3. Insets show the relaxed NT+H geometries; the magenta marker locates C^{*}. For nanotubes we adopt a local axis convention in which p_x and p_y are radial (normal to the tube wall) and p_z is axial; thus, the “out-of-plane” component relevant for $C-H$ bonding corresponds to $p_x + p_y$.

Table S1. Local geometric descriptors at the H adsorption site (C^*) for Dode-NTs with chiralities $(n, 0)$ and $(0, n)$. For each tube, values are reported for the three adsorption sites ($S_1 - S_3$). The pseudo-pyramidalization angle θ_p° quantifies the deviation from planarity at C^*; h_p (Å) is the out of plane height of C^* relative to the least-squares plane through its three nearest C neighbors; and the planarity drop F_p° is defined as $F_p = 360^\circ - \sum_i \alpha_i$, where α_i are the three $C - C^* - C$ bond angles. Larger θ_p and F_p indicate stronger local $sp^2 \rightarrow sp^3$ rehybridization. All values correspond to relaxed geometries with adsorbed H.

Dode-NT	θ_p (°)			h_p (Å)			F_p (°)		
	site1	site2	site3	site1	site2	site3	site1	site2	site3
(4,0)	24.92	24.54	20.83	0.64	0.63	0.53	49.56	66.52	47.67
(7,0)	23.63	23.76	21.16	0.61	0.61	0.54	45.12	63.34	48.74
(10,0)	22.68	23.17	21.52	0.58	0.60	0.55	41.97	61.21	50.02
(0,4)	14.45	26.62	29.42	0.37	0.68	0.74	18.44	69.49	79.01
(0,7)	14.06	26.28	28.10	0.36	0.67	0.70	17.52	68.58	74.11
(0,10)	14.39	25.42	27.41	0.37	0.65	0.69	18.30	66.12	71.54

Table S2. Summary of the main properties of Dode-NTs with (n,0) and (0,n) chiralities.

Property	Dode-NT (n,0)	Dode-NT (0,n)
Curvature energy (E_{curv})	Higher, decreases with n	Lower, decreases with n
Band gap (Gap)	Metallic (Gap $\approx$ 0)	Metallic (even n), semiconducting (odd n)
Young's modulus (DFT)	614-692 GPa	342-398 GPa
Young's modulus (MD)	500-750 GPa	300-430 GPa
Ultimate tensile strength (UTS)	100-120 GPa	80-100 GPa
Binding energy (E_b)	-4.17 to -3.51 eV (S_2 strongest)	-4.31 to -3.98 eV (S_2 strongest)
Adsorption free energy (ΔG_{ads})	-0.62 to 0.08 eV (S_2 near optimum)	-0.69 to -0.12 eV (S_2/S_3 active)
